# Optimization of Microwave-Assisted Green Method for Enhanced Solubilization of Water-Soluble Curcuminoids Prepared Using Steviol Glycosides

**DOI:** 10.3390/foods10112803

**Published:** 2021-11-15

**Authors:** Jin-A Ko, Young-Bae Ryu, Woo-Song Lee, Kashif Ameer, Young-Min Kim

**Affiliations:** 1 Department of Food Science and Technology, Chonnam National University, Gwangju 61186, Korea; jinarhdwn1@naver.com; 2 Functional Biomaterial Research Center, Korea Research Institute of Bioscience and Biotechnology, Jeongeup-si 56212, Korea; ybryu@kribb.re.kr (Y.-B.R.); wslee@kribb.re.kr (W.-S.L.); 3 Institute of Food Science and Nutrition, University of Sargodha, Sargodha 40100, Pakistan

**Keywords:** curcuminoid, microwave, solubilizer, steviol glycoside, optimization, eco-friendly

## Abstract

In this study, the optimization and modeling of microwave-assisted extraction (MAE) of water-soluble curcuminoids prepared using novel steviol glycosides (SGs) was carried out using four independent process variables at varying levels—*X*_1_: microwave power (50–200 W), *X*_2_: stevioside concentration (50–200 mg/mL), *X*_3_: curcumin concentration (20–200 mg/mL), and *X*_4_: time (1–10 min)—in response surface methodology configuration. Moreover, the effects of stevioside, as the most cost-effective natural solubilizer, were also evaluated. The water solubility of curcuminoids increased from 11 to 1320 mg/L with the addition of stevioside as a natural solubilizer. Moreover, microwave heating synergistically with stevioside addition significantly (*p* < 0.05) increased the solubility up to 5400 mg/L. Based on the results, the optimum conditions providing the maximum solubilization of 16,700 mg/L were 189 W microwave power, 195 g/L stevioside concentration, 183 g/L curcuminoid concentration, and 9 min of incubation time. Moreover, MAE of curcuminoids using SGs might render a significant advantage for its wide-scale application to solubilizing the multitude of insoluble functional flavonoids in fruits, plants, and food materials.

## 1. Introduction

Turmeric (*Curcuma longa* L.), as the member of the *Zingiberaceae* family, is found globally in tropical and subtropical regions, including Southeast Asia. Curcuminoids are natural bioactive compounds existing in the form of linear diarylheptanoids [1]. Among the curcuminoids, turmeric has demethoxycurcumin (DMC) and bisdemethoxycurcumin (BDMC), which are natural curcumin congeners/analogues. As major components, the curcuminoid group (also known as diferuloylmethane) in turmeric comprises about 77% of the curcumins (DMC constitutes 17%, and BDMC, 3%) [2,3].

Various published reports pertaining to animal and human studies have endorsed curcuminoids as safe, nontoxic, and tolerable at high doses (≤12 g/day); however, they are not yet approved as therapeutic agents due to limited aqueous solubility and absorption in the human gastrointestinal (GI) tract [1]. Moreover, their utility is limited because of poor water solubility (11 mg/L), relatively low in vivo bioavailability, slow dissolution, and instability in the GI tract under thermal and rapid hydrolysis in alkaline conditions (under pH range of 7.5–9.0) [4]. Curcumin, DMC, and BDMC from *C. longa* had poor solubility in water (11 mg/L), dimethyl sulfoxide (25 g/L), and ethanol (10 g/L), resulting in poor bioavailability [5,6]. Moreover, even with an intake dose of 2 g in humans, curcuminoids exhibit undetectable or extremely low bioavailability (6 × 10^−3^ μg/mL after 1 h in serum) [1,2]. To improve the aqueous solubility and bioavailability of curcuminoids, curcuminoid preparations were constructed in polymeric micelles, liposomes, and cyclodextrin-based delivery systems [5]; self-micro-emulsifying drug delivery was attempted using micelles and nanoparticles [6]; surfactants and co-surfactants were used as well, such as Cremophor RH40 and Kollidon 30. Hydrophobically modified water-soluble polymers exhibit self-assembling properties, and can form micelles in an aqueous phase for curcumin carrying. [7,8] With improved aqueous solubility of curcuminoids, a more than 30-fold increase in bioavailability was achieved after oral administration as compared to that of conventionally used curcumin in a murine animal model [9]. Although synthetic surfactants can effectively increase the solubility of curcuminoids, synthetic surfactants may cause certain undesired characteristics in foods fortified or supplemented with curcuminoids, such as toxicity, quality control issues, and detrimental effects on the targeted delivery of curcumin in its biologically active entrapped form [4]. Moreover, non-biodegradable synthetic surfactants negatively affect the environment and cause several adverse effects on health, such as eye and respiratory irritation and dermatitis [2,10]. Moreover, chemically synthesized curcumin preparations are also available; however, the Joint FAO/WHO Expert Committee on Food Additives (JECFA) only allows use of naturally extracted curcuminoids as food additives [1]. Therefore, researchers worldwide have developed a keen interest in exploring natural, nontoxic, and eco-friendly surfactants of biological origin exhibiting biodegradability, potential for large-scale production, diversity, selectivity, and high-performance throughput under thermal and pH constraints. Thus, in recent years, natural biosurfactants have garnered the attention of researchers worldwide for laboratory-based and industrial applications [6,11].

The type of extraction technique employed exerts a significant influence on the recovery yield of an extracted plant’s active constituents. Traditionally, various trivial techniques involving hydrodistillation, maceration, and solvent extraction have been employed for the recovery of active constituents as powders from plant matrices [12]. However, conventional extraction methods have limited applicability for the targeted recovery of phytochemicals due to the high mass transfer rate owing to low recovery yields, degradation of thermolabile components, large amount of solvent needed, and time and energy consumption [13]. Studies have focused on the exploitation of green extraction methods involving less consumption of nontoxic solvents through modern extraction techniques, such as microwave-assisted extraction (MAE), ultrasound-assisted extraction (UAE), supercritical fluid extraction (SFE), and pressurized liquid extraction (PLE) [12,13,14]. In comparison with other extraction methods, MAE offers several peculiar advantages, such as lower consumption of solvent, process rapidity, multiple sample handling, and convenience of automation [13]. Response surface methodology (RSM), as a multivariate technique, comprising sophisticated mathematical and statistical procedures, is employed for the development, improvement, and optimization of the processes [15].

Various terpene glycosides (steviol monoside, rebaudioside-A, rubusoside, stevioside, geniposide, paenoiflorinm and mogroside V) have been reported to exhibit potential for improving the aqueous solubility of bioactive components of pharmaceutical significance [1]. Steviol glycosides (SGs) are commonly used as natural sweetening compounds and exhibit potential as natural surfactants owing to their amphiphilic structure, because of the presence of a hydrophobic head group in the steviol skeleton and variability in the hydrophilic glycosyl groups connected to C_13_ and C_19_ positions of the molecules [15,16]. Rebaudioside-A’s (Reb-A) surfactant properties have been endorsed by Wolfrum et al. [17]. Moreover, glycosylated SGs may lead to improved bioavailability and solubility of water-soluble components [18]. Previously published studies have indicated that rubusoside is an effective solubilizer, and a 10% rubusoside aqueous solution (*w*/*v*) leads to enhanced solubility of curcumin (0.6–2.3 mg/mL), but its industrial application is not cost-effective [19,20]. Curcuminoids are practically reported to be insoluble in acidic mediums and may be hydrolyzed into feruloylmethane, ferulic acid, and yellowish-brown condensation products. In aqueous phases of SGs, curcuminoids exhibited stability over a pH range of 2–10 for 2 h (at 80 °C) and 1 h (at 120 °C) [1]. Therefore, water-soluble curcuminoids need to be investigated with various SGs.

In our present study, the solubility and stability of curcuminoids in the presence of steviol glycosides were evaluated, and then optimization and modeling of MAE of water-soluble curcuminoids prepared using stevioside glycosides was carried out using four independent process variables at varying levels—*X*_1_: microwave power (50–200 W), *X*_2_: stevioside concentration (50–200 mg/mL), *X*_3_: curcumin concentration (20–200 mg/mL), and *X*_4_: time (1–10 min)—under the RSM-based Box–Behnken design (BBD) configuration. Moreover, the effects of SGs, including stevioside, as the most cost-effective natural solubilizer, were also evaluated.

## 2. Materials and Methods

### 2.1. Materials

Five commercial stevia extracts and derivatives (enzymatically-modified stevioside, SWETA, ML01, SWETA75, and stevioside) were purchased from Daepyung Co. Pvt. Ltd. (Seongnam, Korea). Curcuminoids, stevioside, steviol, rubusoside, and Reb-A were purchased from Sigma–Aldrich Co. (St. Louis, MO, USA). Other SGs, including steviol monoglucoside, steviolbioside, and steviol mono-glucosyl ester were prepared in our laboratory as previously described [19,21]. Turmeric powder was purchased from a local market. Curcuminoids (≥94%) containing ≥80% curcumin (PubChem CID: 969516), DMC (PubChem CID: 5469424), and BDMC (PubChem ID: 5315472) were purchased from Sigma as standard. Glucosyl stevioside was synthesized and isolated using *Leuconostoc* glucansucrases [22,23,24]. All reagents and chemicals were of analytical grade and were purchased from Sigma–Aldrich Co.

### 2.2. Preparation and Solubilization of Water-Soluble Curcuminoids by Stevia Extracts

Curcuminoids were solubilized by the addition of four commercial stevia extracts (SWETA, ML01, SWETA75, and stevioside) and enzymatically-modified stevioside, and the solubilized curcuminoids were subsequently quantified with different retention times in HPLC. Then, stevioside was used to solubilize the curcuminoids for further study. The effect of water concentration to extract curcuminoids was investigated. Each Ste, RebA, or SG at 10% (*w*/*v*) was mixed with 30% (*w*/*v*) of turmeric powder followed by the addition of 0–80% (*v*/*v*) of water in ethanol. The mixture in an ethanol solution was vortexed for 15 min and centrifuged at 12,000× *g* for 10 min (SupraR30, W × D × H: 710 × 1000 × 1260 mm, Hanil Scientific Inc., Gimp, Korea). The supernatant was transferred to a new tube and ethanol was evaporated [10]. The resulting powders were dissolved in water, centrifuged at 12,000× *g* for 10 min, and filtered through a 0.20 μm membrane (Agilent, Santa Clara, CA, USA).

Weighing of the appropriate amounts of each steviol glycoside and curcuminoids was carried out, and they were put into 50 mL glass bottles. Furthermore, the suspension of curcuminoid–SG was subjected to heating by exposure to microwave irradiation in a microwave-assisted extractor (CEM, Matthews, NC, USA) at 100 W and 25 °C. Prior to conducting chromatographic analyses as per the reported method of Nguyen et al. [1], the filtration of all samples was performed using a 0.45 μm nylon syringe filter (Millipore, Billerica, MA, USA). The quantitative concentration determination of curcuminoids and SGs was performed by employing series of standard solutions of curcuminoid in methanol with concentrations ranging from 0.15 to 10 μg/mL according to the reported method [1]. Chromatographic separation for quantitative analysis was carried out using an Agilent 1200 liquid chromatographic system (Agilent Technologies, Palo Alto, CA, USA) equipped with an autosampler, quaternary HPLC pump, degasser, ZORBAX SB-C_18_ column (5 μm, 4.6 × 150 mm), and UV detector (VWD) set at 210 and 260 nm. The mobile phase for HPLC consisted of solvent A, which was 0.1% trifluoroacetic acid in water, and solvent B, which was 0.1% trifluoroacetic acid in acetonitrile. The solvent gradient was as follows (relative to solvent A): 0 min, 80% A; 10 min, 75% B; 20 min, 65% B; 30 min, 50% B; 40 min, 40%; 50 min, 30%; 60 min, 0% B; 0 min, 20%; 10 min, 25% B; 20 min, 35% B; 30 min, 50%; 40 min, 60%; 50 min, 70%; and 60 min, 100%. The column temperature was maintained at 30 °C with an injection volume of 10 μL at a flow rate of 1.0 mL/min.

### 2.3. Determination of Surface Morphology

A field emission transmission electron microscope (FE-TEM, FEI Tecnai G2 F20, Noord-Brabant, The Netherlands) equipped for X-ray energy dispersive spectroscopy at a working voltage of 200 kV was used to determine the surface morphology of the curcuminoid–stevioside nanoparticles. Briefly, the depositing drop comprising of 5 μL of aqueous curcuminoid–stevioside solution was placed on a copper grid (BAL-TEC, Los Angeles, CA, USA) at room temperature (20–25 °C). The sample was allowed to stand for 15–30 s and excessive solution was removed by blotting.

### 2.4. Particle Size Measurement

The particle size and distribution were measured with an acoustic spectrometer (DT 1200, Dispersion Technology Inc. Bedford Hills, NY, USA) according to method of Sun et al. [25]. The aqueous solution of curcuminoids in 10% (*w*/*v*) stevioside was transferred onto clean cells maintained at 25 °C by using a circulating water bath. Each sample was run five times at a 90° angle, and the duration of each run was 180 s. A calibration program provided by the instrument manufacturer was used to calculate the particle size distribution from the obtained attenuation spectra.

### 2.5. Preparation of Aqueous Solutions of Curcuminoid–stevioside and Quantification

Aqueous solutions of curcuminoid–stevioside containing 1%, 2%, 4%, and 10% (*w*/*v*) stevioside were prepared according to the aforementioned procedures, stored at room temperature, and protected from light (three replicates). The concentrations of curcuminoids in the solutions were measured after 0, 2, 4, 8, 12, and 24 h by HPLC after sample preparation as per the reported method [1]. As a control, an aqueous solution of only stevioside (10% *w*/*v*, three replicates) was prepared in the same way, and the concentrations of stevioside in the solutions were measured after 0, 2, 4, 8, 12, and 24 h by HPLC after sample preparation.

### 2.6. Experimental Design

Preliminary screening experiments were performed in order to determine appropriate ranges of independent variables, such as microwave power, stevioside concentration, curcuminoid concentration, and incubation time. Choosing the independent practical variables was carried out on the basis of literature reviews [26,27]: microwave power (*X*_1_: 50–200 W), stevioside concentration (*X*_2_: 50–200 mg/mL), curcuminoid concentration (*X*_3_: 20–200 mg/mL), and incubation time (*X*_4_: 1–10 min). *X*_1_ was varied over a range of 50–200 W, while *X*_2_*, X*_3_, and *X*_4_ were kept constant at 125 mg/mL, 110 mg/mL concentrations of stevioside and curcuminoids, and 6 min of incubation time, respectively. Similarly, *X*_2_ was varied from 50–200 mg/mL, while *X*_1_*, X*_3_, and *X*_4_ were fixed at 125 W, 110 mg/mL, and 6 min, respectively. Likewise, *X*_3_ was also evaluated by being varied over a range of 20–200 mg/mL, while *X*_1_, *X*_2_, and *X*_4_ were kept constant at 125 W, 125 mg/mL, and 6 min, respectively. Moreover, evaluation of *X*_4_ was performed by varying it over the range of 1–10 min with fixed levels of *X*_1_*, X*_2_, and *X*_3_ at 125 W, 125 mg/mL, and 110 mg/mL. The optimization experiments were carried out according to a BBD configuration with four factors and three levels with the aim of optimizing the curcuminoid solubilization procedure using Design Expert 8.0.1 software (Stat-Ease Inc.; Minneapolis, MN, USA). The design included six replicates at the central point, which were utilized in the fitting of a second-order response surface. In total, 27 experimental runs (comprising 16 factorials, 8 axial, and 3 center points) were performed using four independent variables at various levels: microwave power (*X*_1_: 50–200 W), stevioside concentration (*X*_2_: 50–200 mg/mL), curcuminoid concentration (*X*_3_: 20–200 mg/mL), and incubation time (*X*_4_: 1–10 min). The optimization was conducted using a desirability function to determine the effects of *X*_1_, *X*_2_, *X*_3_, and *X*_4_ on curcuminoid solubilization. Independent variables with units in terms of coded notations were provided along with experimental ranges. The coding of the variables was carried out as per the following equation [12]:

(1) xi=Xi−XcpΔXi where i=1, 2, 3,…, k

where *X_i_* represents a dimensionless value of the independent process variable; and *x_i_*, *X_cp_*, and Δ*X_i_* represent the real value, real value at the central point, and rate of change with respect to variable i, respectively. After the completion of 27 experimental runs, fitting of the second-order quadratic model to the response data as a function of independent variables was carried out through Design Expert software as per the following equation:

(2) $$Y=\beta_{0}+\sum_{i=1}^{n}\beta_{i}X_{i}+\sum_{\begin{array}{c} i=1\\ j>1 \end{array}}^{n-1}\sum_{j=2}^{n}\beta_{ij}X_{i}X_{j}+\sum_{i=1}^{n}\beta_{ii}X_{i}^{2}+\epsilon$$

where *Y* represents the response variable; $\beta_{0}$, $\beta_{i}$, $\beta_{ii}$, and $\beta_{ij}$ represent regression coefficients for intercept, linear, quadratic, and interaction terms, respectively; $X_{i}$,$\;X_{j}$, and $X_{ij}$ represent coded independent variables; and ε represents random error. Moreover, each variable was analyzed for its effect on the target response. Analysis of variance (ANOVA) was performed by the software, and model significant terms were determined. With the initial model, step-wise selection was employed to remove statistically non-significant terms (*p* > 0.05), and the refitting of experimental data was carried out to yield the final model [12]. Interaction effects of independent variables on the target response were elucidated through response surface plots derived from the developed model.

### 2.7. Recombinant Influenza Virus Neuraminidase Inhibition Assay

The inhibitory effect on rvH1N1 neuraminidase (A/Bervig Mission/1/18, rvH1N1) was measured using the fluorometric method developed in our previous study [28]. Curcumin was dissolved in MeOH or stevioside. A reaction mixture containing sialidase (0.025 pg/mL) and 800 μM methylumbelliferyl-α-d-*N*-acetyl-neuraminic acid, together with the curcuminoids, was incubated in 50 mM Tris-HCl buffer (pH 7.5) at 25 °C. The emission at 445 nm was determined for excitation at 365 nm using the FLx 800 system (BioTeck Instrument Inc. Winooski, VT, USA). At least three experiments were performed for each sample. The mean neuraminidase activity of the untreated control was set to 100%, and the concentration required to reduce the neuraminidase activity of the control by 50% was calculated.

### 2.8. Statistical Analysis

The results of SGs, such as stevioside, Reb-A, rubusoside, steviol mono-glucoside, steviol monoglucosyl ester, steviol, and curcuminoids, are each expressed as the mean ± standard deviation of three identical experiments. One-way ANOVA was performed using GraphPad Prism software version 5.0 (GraphPad Software Inc.; San Diego, CA, USA). Statistical significance was determined as *p* < 0.05.

## 3. Results and Discussion

### 3.1. Extraction and Solubilization of MAE Water-Soluble Curcuminoids Using SGs

In our previous studies, a novel stevioside-specific β-glucosidase, SSGase, was isolated from *Aspergillus aculeatus* [19,24]; and a steviol-producing β-glucosidase, SPGase, isolated from *Penicillium decumbens*, was screened, purified, and characterized.^16^ In addition, for SSGase, the conditions of rubusoside production were optimized by RSM using four important factors (stevioside concentration, enzyme activity, temperature, and pH). SPGase was selected to produce steviol from stevioside via steviolbioside, rubusoside, steviol mono-glucosyl ester, and steviol mono-glucoside. Moreover, the parameters required for the maximum production of steviol (30 mM, 64% yield) from stevioside by RSM were described by Ko et al. [23] as 47 mM stevioside and 43 μL of SPGase at pH 4.0 and 55 °C. These SGs or stevia extracts were used to screen the solubilized curcuminoid solution. The tinge of yellow color became darker with corresponding increases in SG concentrations, indicating that more curcuminoids had dissolved in the solution. Curcuminoids were solubilized to various degrees, with solubility ranging from 540 to 1620 mg/L, depending on the glucose moiety and the type of SG used (Table 1). Among the tested SGs, the solubility values of curcuminoids were 1620 and 1420 mg/L in the 10% (*w*/*v*) rubusoside and stevioside solutions, respectively. Water-soluble curcuminoids were obtained at a 1.4-fold lower rate than the value of 2318 mg/L reported by Zhang et al. [29]. Given the similar solubility values, stevioside was selected as a good solubilizer because its cost was 10 times lower compared to that of rubusoside [19]. HPLC analyses determined that the concentrations of dissolved curcuminoids in curcuminoid–stevioside were 55, 270, 590, 820, 1249, and 1420 mg/L for the stevioside concentrations of 1%, 2%, 4%, 6%, 8%, and 10% (*w*/*v*), respectively. As evident from the decreasing order of curcuminoid solubility—rubusoside = stevioside > Reb-A > α-glucosyl stevioside = steviol mono-glycoside > steviol = steviol mono-glucosyl ester = steviolbioside—the balance between the hydrophilic glucosyl residue and each hydrophobic steviol backbone is an important factor for curcumin solubilization. As far as dissolution is concerned, it not only encompasses the state of complete dissolution of curcuminoid–stevioside in the aqueous phase, but also underpins the state of being solubilized by micelles or similar substances, and a state of uniform dispersion of a liquid in binary solutions or an aqueous phase. It might be implied by the results that the the enhanced solubility of curcuminoid–stevioside owes to complex formation between curcuminoids and SGs without clear formation of covalent linkages between SGs and curcuminoids, which resulted in the formation of physically complex structural configurations through intermolecular linkages [1].

During pre-experiments, various physical treatments were employed to improve the solubility of water-soluble curcuminoids. Among the physical treatments tested (microwave, ultrasonication, electron beam, and autoclaving), exposure to microwave treatments yielded higher concentrations of water-soluble curcuminoids (9270 mg/L) than ultrasonication (1120 mg/L) or autoclaving (1530 mg/L). However, treatment by electron beam irradiation (10‒200 kGy) did not exert any significant (*p* > 0.05) effect (data not shown). When assessing the water solubility of curcuminoids at various pH levels, it was evident that curcuminoids exhibited poor degrees of solubility in water at acidic and physiological pHs, and were hydrolyzed rapidly in alkaline solutions. The curcuminoids were kept at room temperature for 15 min in 40 mM Britton–Robinson buffer (pH 3.01–10.0); then an HPLC assay and sample preparation were carried out, which resulted in a high degree of curcumin solubility and improved stability at pH 5.0–7.0. In order to evaluate the stability in 10% (*w*/*v*) SGs, the water-soluble curcuminoids were analyzed by HPLC for 1 month. Curcuminoids’ solubility was found to remain stable over a 24 h period at 25 °C. Compared with first-day curcuminoids in 10% stevioside, they remained soluble to the maximum extent of 84.5% for 1 month at 25 °C (Table 2). These results show that water-soluble curcuminoids are highly stable at room temperature. Microwave treatment with stevioside in a synergistic manner can rapidly and effectively lead to the generation of high concentrations of water-soluble curcuminoid solutions. The solubilized curcuminoid had a distinct yellow color that was distinguishable from that of colorless steviol glycosides. A curcuminoid solubility of 9270 mg/L was achieved after exposure to microwave irradiation, which was four times higher than of the solubility value reported by Zhang et al. [29]. Microwave irradiation may accelerate micelle formation between steviol glycosides and curcuminoids by heating them using the appropriate power and exposure time. The microwave irradiation-induced acceleration of micelle formation might be because of reaction selectivity and the transglycosylation phenomenon causing the generation of reducing sugar intermediates followed by disproportionation of steviol from SG [20]. In order to achieve better solubility, transglycosylation is regarded as an important procedural phenomenon that leads to structural modifications and derivatizations that result in enhanced biological activities and improved solubility. Moreover, heating in the case of microwave irradiation is usually dependent on the nature of the sample, the solvent employed, and the irradiation power level mediated through the ionic conduction and dipole rotation (also known as dipole polarization) of the microwaves [20,30]. In the absence of ions, water dipoles are found in the free form and may experience dipole rotation due to microwave radiation-induced oscillation. Hence, this phenomenon led to an increasing tendency in the overall kinetic energy of the system, which consequently enhanced the temperature of the water molecules in the aqueous phase, and hence improved the solubility, owing to increased mass transfer of curcuminoids. It may also be implied that the increased temperature could be because of ionic conduction and dipole rotation of the molecules, which caused heat generation by friction and collisions with the water molecules [13,31]. In the case of ionic conduction, ions migration takes place when they are subjected to the influence of a electromagnetic field, and ions existing in the solution which offer resistance to flow may lead to the generation of friction-induced heat. Dipole rotation in MAE encompasses realignment of the dipoles under the influence of an applied electromagnetic field. This forced movement of dipoles results in molecular friction, and the solution is subsequently heated [14]. The high curcuminoid solubilities achieved in the stevioside solutions and microwave treatments may be comparable to or exceed those obtained with cyclodextrin, [5] or with surfactants, such as Cremophor RH40 or Kollidon 30 [7], and liposomes [8]. The increased solubility could be due to increased mass transfer and the effect of solid content on the recovery of bioactive compounds mediated through the concentration gradient occurring between the plant matrix and solvent when subjected to electromagnetic irradiation during MAE [14]. Previously, the use of organic solvents, such as *n*-hexane, isopropanol, ethyl acetate, ethanol, methanol, and acetone, was reported with Soxhlet, UAE, zone-refining, and dipping techniques, followed by crystallization. However, curcuminoids are classified as hydrophobic polyphenols with poor aqueous solubility [1,32]. Moreover, the applicability of food and pharmaceuticals’ curcuminoids is also restricted, owing to very low bioavailability and instability in both in vitro and in vivo conditions [1]. Furthermore, Wake et al. [33] have compared the extraction efficiency of various extraction methods, such as MAE, UAE, and SFE with that of conventional Soxhlet extraction in terms of extractions yields. It was reported by the authors that MAE exhibited significant potential with the highest recovery of curcumin from rhizome matrices of powdered *C. longa*. Several studies have reported an increase in the bioavailability of bioactive compounds using SGs; however, the exact mechanism is still unknown. Stevioside, Reb-A, and other SGs are chemically classified as aglycon glycosides based on aglycon’s central carbon-skeleton, steviol. Stevioside, Reb-A, and other SGs consist of a D-glucose groups configured at C_19_ [29]. Specifically, stevioside possesses di-glucosyl, whereas Reb-A comprises a tri-glucosyl sugar moiety at C_13_. Stevioside, Reb-A, and other SGs exhibit comparatively high aqueous solubility; however, the steviol present in the structural configuration of SGs is hydrophobic in nature at its central position [10]. Nguyen et al. [1] has also reported that the water-soluble curcuminoids yielded from turmeric powder showed a decreasing solubility tendency when the number of glucose units affixed to steviol at C_13_ was correspondingly increased. Instead of stevioside, when other SGs were used for solubilization of water-soluble curcuminoids, the relative concentration of curcumin showed slightly decreasing trend. Therefore, it was suggested in the literature that stevioside should be used in synergy with Reb-A and other SGs which might be able to improve curcuminoids’ stability at different pH values [1,34]. However, the exact mechanistic details pertaining to the solubilization of hydrophobic components require further investigation.

### 3.2. Microstructural Features of Water-Soluble Curcuminoids

**Figure 1:**
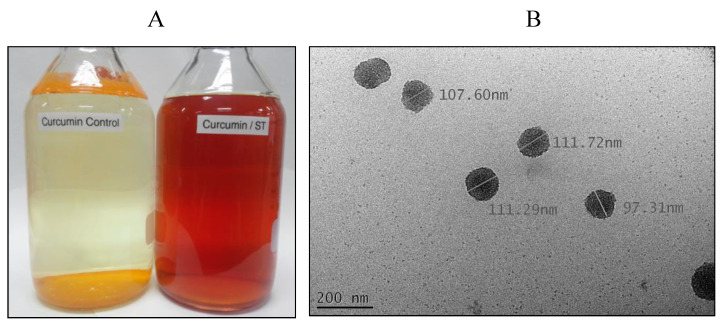
(**A**) Curcuminoids dissolved in water and in 10% (*w*/*v*) stevioside. (**B**) Field-emission transmission electron microscope (FE-TEM) image of curcuminoid–stevioside nanoparticles in the 10% (*w*/*v*) stevioside.

TEM micrographs of aqueous curcuminoid solution and the solubilized curcuminoid–stevioside solution are shown in Figure 1. Apparent colors of the curcuminoids dissolved in water and in 10% (*w*/*v*) stevioside are demonstrated in Figure 1A. The particle morphology of the curcuminoid–stevioside nanoparticles in 10% (*w*/*v*) stevioside solution is shown in Figure 1B. The particles appear to have been mono-dispersed, which implies that all solubilized particles were found in the dispersed phase with uniform size. As colloidal properties rely on particle characteristics—such as structure, size, and shape—and particle uniformity, mono-dispersed particles are pertinent, as they may exert a significant influence on the rate of mass transfer [25]. Moreover, the color of the curcumin aqueous solution was not homogenous, as most of the curcuminoids remained on top of the water. In contrast, the curcuminoids solubilized in 10% (*w*/*v*) stevioside were deep brown in color. The average size of the curcuminoid–stevioside nanoparticles in the 10% (*w*/*v*) stevioside solution was 106 ± 34 nm. Figure 1B shows FE-TEM images of the curcuminoid–stevioside water solution, in which numerous dark spots, believed to be the nanoparticles formed from curcuminoids and stevioside, were observed. The TEM images show that curcuminoid–stevioside nanoparticles exhibited a spherical morphology (Figure 1B) and that the particle size (<150 nm) resembled the results that were obtained from the acoustic spectrometry. The amphiphilic structure of stevioside allows it to form micelles with water molecules in the aqueous phase. SGs (stevioside and Reb-A) exhibit the presence of a d-glucose group with a fixity at C_19_. Di-glucosyl and tri-glucosyl sugar moieties have been reported for stevioside and Reb-A with affixations at C_13_ [1]. Usually, stevioside and Reb-A have high degrees of solubility in aqueous solutions, whereas steviol exhibits less, due to the hydrophobicity at the center of its structural configuration. With respect to the yield recoverys of water-soluble curcuminoids, an increasing tendency in glucose units linked to steviol at C_13_ has been reported to exert a negative effect on the yields of water-soluble curcuminoids [1,35]. Similarly to the findings reported by Zhang et al. [27], we found that the stevioside molecule had a bolaform amphiphile—a hydrophobic ring in the center and two hydroxyl groups on each end. Stevioside is a diterpene ent-kaurene glycoside that possesses a hydrophobic steviol backbone and hydrophilic glucosyl and sophorose residues. Thus, it might be inferred that the stevioside molecules can self-assemble to minimize the exposure of their hydrophilic groups to water, which is consistent with the behavior exhibited by other bolaform amphiphiles [36]. A recent study demonstrated that, compared to larger nanoparticles, nanoparticles that are smaller than 200 nm are more efficiently distributed in tumors [37]. In a drug/ingredient delivery system, particle size greatly influences the pharmacokinetics of the drug or food ingredient circulation time, adsorption, and distribution [8,38]. Kroyer [34] has also reported that stevioside imparts a protective effect against delayed degradation of curcuminoids induced by ascorbic acid. Moreover, curcuminoid–stevioside nanoparticles are small, and a decrease in size leads to a corresponding rise in surface area to volume ratio. A proportionally higher surface area results in more interactions of particles with the solvent/extractant, hence causing a higher degree of solubility. Aqueous-soluble curcuminoids exhibit a fairly narrow size distribution owing to lower polydisperity index of <0.2 [1]. Furthermore, it was also endorsed by Sasaki et al. [9] that curcuminoids having a size distribution ranging from 100 to 1000 nm and a mean particle size of 190 nm exhibited improved bioavailability—up to 27-fold—in human serum as compared to curcumin powder. This implies that the solubility and bioavailability improvements to aqueous-soluble curcuminoids extracted through SGs have great potential for future applications.

### 3.3. Optimization of Conditions for Water-Soluble Curcuminoids

RSM was used to study the interactions of various factors in relation to curcuminoid solubilization using Design Expert 8.0.1 software. All experimental runs were carried out in accordance with BBD-based configuration for optimizing curcuminoid solubilization as a function of microwave power (50–200 W), stevioside concentration (50–200 mg/mL), curcuminoid concentration (20–200 mg/mL), and incubation time (1–10 min). To analyze the adequacy and significance of the developed model, ANOVA was performed [39], and the results are tabulated in Table 3 in terms of experimental and predicted findings for curcuminoid solubility [12,15].

The effects of the four independent variables were also elucidated, where *X*_1_, *X*_2_, *X*_3_, and *X*_4_ are the symbols for microwave output (W), stevioside concentration (mg/mL), curcuminoid concentration (mg/mL), and incubation time (min), respectively. Target response values and the second order quadratic model were subjected to analysis through multiple linear regression (MLR) to achieve a good fit. Curcuminoid solubility is expressed in terms of the following regression Equation (3):

*Y* = 4.94 + 2.21*x*_1_ + 3.42*x*_2_ + 1.33*x*_3_ + 1.29*x*_4_ + 1.88*x*_1_*x*_2_ + 2.08*x*_1_*x*_3_ + 0.40*x*_1_*x*_4_+ 1.31*x*_2_*x*_3_ + 0.91*x*_2_*x*_4_ + 0.75*x*_3_ *x*_4_ − 0.61*x*_1_^2^ + 0.35*x*_2_^2^ + 0.37*x*_3_^2^ − 1.55*x*_4_ ^2^ (3)

Results show that a high degree of significance was exhibited by the developed model*,* with lower probability values (*p* < 0.0002), high determination coefficient (R^2^) values, and adequate precision of 11.541, indicating that this model can be used to navigate the design space. The higher precision and R^2^ values endorse the validity of the developed model. Furthermore, the significance of the model was determined using the lack of fit and *F*-value. Lack of fit was another parameter used to authenticate the model’s validity, and the lack of fit was non-significant (*p* > 0.05), as shown in Table 4. The *F*-value was 22.24, suggesting that it was not significant, and there was only a 0.01% chance that this result would occur due to noise [40].

**Figure 2:**
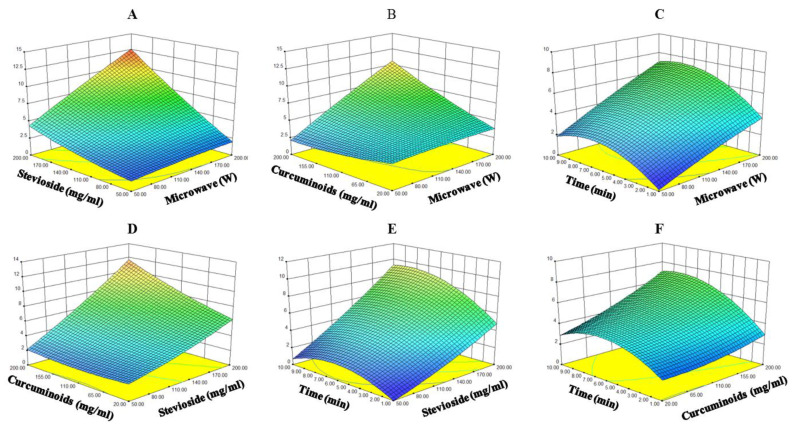
Response surface and contour plots of curcuminoid solubility with stevioside. Mutual interactions between stevioside concentration and microwave level (**A**), curcumin concentration and microwave level (**B**), time and microwave level (**C**), curcumin concentration and stevioside concentration level (**D**), time and stevioside concentration level (**E**), and time and stevioside concentration. Other parameter levels (**F**). All other parameters, except for the two in each figure, were at their respective zero levels.

Thus, the fitted model was able to explain 92% of the variability, which suggests that a close correlation existed, because the *R*^2^ value is higher than 0.8. Based on this model, the predicted response for curcuminoid solubilization was 17,065 mg/L, whereas the observed experimental value was 16,700 mg/L at 195 g/L of stevioside and 183 g/L of curcuminoid at 189 W for 9 min. Moreover, the predicted and observed values also showed the fair match for curcuminoid solubilization. Linear, cross-product, and interaction effects were also elucidated by the ANOVA results, and it was evident that all effects significantly (*p* < 0.05) influenced the solubility. The obtained solubility was 7.2-fold higher than that reported by Zhang et al. [29]. To the best of our knowledge, this stevioside-based microwave-assisted method helped us to achieve the highest concentration of dissolved curcuminoids reported to date, which was evident from the central points of the corresponding response surface and contour plots shown in Figure 2. Response surface plots for the target response exhibit a high degree of convexity with a well-defined rising ridge, and the highest solubility can be observed around the mid-point regions. This phenomenon is explicable with respect to the influential effects of microwave power, stevioside and curcuminoid concentrations, and incubation time. We believe that this stevioside-based microwave-assisted method may also be applied to other water-insoluble bioactive compounds that are used in food and pharmaceutical industries. However, the developed model exhibited a non-significant lack of fit, which implied well-fitting of the experimental data and endorsed the model’s validity to predict the response variable under any combination of independent variables according to BBD configuration. A high *R*^2^ value (0.9206) was obtained from the MLR analysis (Table 4).

### 3.4. Stability of the Conditions for Water-Soluble Curcuminoids Using Stevioside

With regard to the stability of curcuminoids dissolved using stevioside, it has been widely recognized that differences in temperature need to be taken into consideration when planning stability studies to determine the shelf-lives of drugs and other bioactive compounds used in the pharmaceutical and food industries [1]. After preparation, aqueous solutions of water-soluble curcuminoids in 10% (*w*/*v*) stevioside (three replicates) were subjected to storage at ambient room temperature and protected from light for 24 h. The results demonstrated that curcuminoids solubilized in stevioside and rubusoside solutions were highly stable at room temperature. However, gradual changes in the stability of the curcuminoids solubilized in rubusoside or Reb-A occurred over time. Results showed that rubusoside does not have greater stability as compared to stevioside. Aqueous solutions of curcuminoids prepared using rubusoside and Reb-A did not exhibit good stability when subjected to long-term storage. In contrast, α-glucosyl stevioside showed very good stability over the long-term storage interval. These results are in agreement with the findings of Nguyen et al. [1], who reported the effects of pH on the stability of water-soluble curcuminoids extracted with stevisoide and Reb-A, and found over 84% stability when subjected to storage conditions with pH values from 6 to 7.

### 3.5. Neuraminidase Inhibitory Activity

Curcumin is an effective antiviral material with the double effect of inhibiting viral infections and inhibiting hemagglutination activity [41,42]. To test water-soluble curcuminoids with stevioside, we used an rvH1N1 neuraminidase inhibitory assay. Generally, the addition of methanol to the reaction mixture decreased neuraminidase activity because of structural changes to the enzyme. Water-soluble curcuminoids with stevioside acted as effective anti-neuraminidase agents without any decrease in enzyme activity. Lineweaver–Burk and Dixon plots confirmed that curcuminoids in stevioside exhibited noncompetitive inhibition characteristics against the influenza virus neuraminidase, as previously reported by Dao et al. [41]. Methanol would not be therapeutically useful if the solubilized curcuminoids did not interact with neuraminidase. However, the neuraminidase inhibitory activity of the water-soluble curcuminoids with stevioside (IC_50_, 3.5 ± 0.15 μg/mL) was identical to that of curcuminoid–methanol (IC_50_, 3.6 ± 0.2 μg/mL), regardless of the solubilizing agent. Therefore, the water-soluble curcuminoids with stevioside were equally bioavailable and efficacious when compared with curcuminoids solubilized in methanol. This finding demonstrates that the bioactivity of curcuminoids can be completely maintained when they are solubilized in the presence of stevioside.

## 4. Conclusions and Future Perspectives

Curcuminoids in turmeric have been in use for many applications in food processing as pigments and in traditional and alternative medicines. For using curcuminoids in various applications, water solubility is one of the most significant determinants for solubilization. In this study, we demonstrated a novel solubilization method for poorly soluble curcuminoids using steviol glycosides. Microwave-assisted extraction (MAE) dramatically enhanced the solubility of the curcuminoids. Using 195 g/L of stevioside and 183 g/L of curcuminoid at 189 W microwave power for 9 min, we achieved a solubility of 16,700 mg/L for curcuminoids. This result demonstrates that the bioactivity of curcuminoids can be completely maintained when they are solubilized in the presence of stevioside or rubusoside. Although numerous attempts have been made to solubilize curcuminoids by using a self-microemulsifying drug delivery system, the major advantage of stevioside as a safe solubilizing agent is that curcuminoid–stevioside can be easily applied to animal studies and translational investigations in humans. Moreover, extracting curcuminoids via MAE using steviol glycosides might have a significant advantage for solubilizing the multitude of insoluble functional flavonoids in fruits, plants, and food materials. Keeping in view the findings of the current study, future investigations will evaluate water-soluble curcuminoids in animal and human models. During routine analyses at the laboratory scale, MAE is used widely as a sample preparation technique, and it is for laboratory-scale extractions of bioactive constituents from plant matrices and biological samples. With regard to scaling-up to the industrial level, the main obstacle is achieving the required frequency of microwaves. For large-scale extractions at the industrial level, a lower MAE frequency of 915 MHz is desirable to allow penetration of electromagnetic waves to a greater extent. The main problem in this scenario would be slower warming of the solution matrix and irregular heating of the samples. However, while a solution to this problem is to use high frequency microwaves (2.45 GHz), the maximum allowable sample thickness should be 2 cm, as at a higher frequency of 2.45 GHz, the waves will have a smaller wavelength and subsequently poorer penetration into the samples. The other factor is cost-effectiveness. Industrial scale-up of MAE is possible, and microwave-assisted large-scale extractors and reactors have already been employed in some industries; however, the cost factor of MAE application needs to be considered. In this regard, further research is needed, and the priorities are as follows: (1) the need to carry out thorough investigations of modern extraction methods at pilot scales; (2) mechanistic studies should be taken into account for enhanced understanding of the mechanisms involved in the extraction kinetics of green extraction methods; (3) designs and processes of green extraction methods should be optimized for removal of technical barriers in future scaling-up of extraction processes to the industrial level.

## Figures and Tables

**Table 1:** Solubility of curcuminoids in the presence of steviol glycosides.

Steviol Glycoside (10%-*w*/*v*)	Curcumin (mg/mL)		Demethoxycurcumin (mg/mL)		Bisdemethoxycurcumin (mg/mL)		Total Curcuminoids (mg/mL)		
	NT	Microwave	NT	Microwave	NT	Microwave	NT	Microwave	Ratio Microwave /NT (fold)
S	-	-	-	-	-	-	-	-	-
SME	-	-	-	-	-	-	-	-	-
SteB	-	-	-	-	-	-	-	-	-
SMG	0.46 ± 0.1	2.52 ± 0.15	0.04 ± 0.01	0.88 ± 0.1	0.04 ± 0.01	0.52 ± 0.1	0.54 ± 0.1	3.92 ± 0.5	7.3
R	1.31 ± 0.1	6.19 ± 0.15	0.08 ± 0.01	1.69 ± 0.15	0.23 ± 0.08	1.17 ± 0.2	1.62 ± 0.2	9.28 ± 0.4	5.7
ST	1.02 ± 0.1	6.29 ± 0.2	0.05 ± 0.01	1.64 ± 0.2	0.05 ± 0.01	1.34 ± 0.15	1.22 ± 0.2	9.27 ± 0.3	7.6
STG	0.51 ± 0.2	2.72 ± 0.3	0.05 ± 0.01	0.83 ± 0.1	0.04 ± 0.01	0.69 ± 0.2	0.61 ± 0.1	4.24 ± 0.6	7.0
Reb-A	0.77 ± 0.2	3.67 ± 0.3	0.06 ± 0.01	0.8 ± 0.2	0.03 ± 0.01	0.28 ± 0.2	0.86 ± 0.2	4.75 ± 0.5	5.5

Each curcuminoid was quantified by high-performance liquid chromatography, as described in the methods. NT, not treated by microwave; S, steviol; SME, steviol mono-glucosyl ester; SteB, steviolbioside; SMG, steviol mono-glucoside; R, rubusoside; ST, stevioside; STG, α-glucosyl stevioside; Reb-A, rebaudioside.

**Table 2:** Stability of the conditions for dissolving curcuminoids using various steviol glycosides.

Steviol Glycoside (10%-*w*/*v*)	Soluble Curcuminoids (10 mg/mL)		
	1 Day	1 Week	1 Month
Steviol	-	-	-
Steviol mono-glucoside	-	-	-
Steviolbioside	-	-	-
Steviol mono-glucosyl ester	2.52 ± 0.05	2.39 ± 0.05	1.81 ± 0.1
Rubusoside	6.19 ± 0.15	5.89 ± 0.1	3.81 ± 0.15
Stevioside	6.29 ± 0.15	6.09 ± 0.15	5.32 ± 0.15
α-Glucosyl stevioside	2.72 ± 0.05	2.69 ± 0.1	2.42 ± 0.1
Rebaudioside A	3.67 ± 0.05	2.21 ± 0.1	1.31 ± 0.1

Each curcuminoid was quantified by high-performance liquid chromatography, as described in the methods.

**Table 3:** Box–Behnken design matrix for the experimental design and the predicted responses for curcuminoid solubility.

Exp. No.	Coded Value				Actual Value				Solubility (mg/mL)	
	*x*_1_	*x*_2_	*x*_3_	*x*_4_	Microwave Power (W)	Stevioside Conc. (mg/mL)	Curcumin Conc. (mg/mL)	Incubation Time (min)	Experimental	Predicted
1	−1	−1	0	0	50	50	110	6	0.66 ± 0.01	1.38
2	1	−1	0	0	200	50	110	6	2.32 ± 0.01	2.04
3	−1	1	0	0	50	200	110	6	3.91 ± 0.1	4.45
4	1	1	0	0	200	200	110	6	13.11 ± 0.3	12.65
5	0	0	−1	−1	125	125	20	1	2.55 ± 0.1	1.88
6	0	0	1	−1	125	125	200	1	2.19 ± 0.1	3.04
7	0	0	−1	1	125	125	20	10	3.56 ± 0.1	2.97
8	0	0	1	1	125	125	200	10	6.2 ± 0.2	7.13
9	−1	0	0	−1	50	125	110	1	1.67 ± 0.05	0.11
10	1	0	0	−1	200	125	110	1	2.3 ± 0.1	3.75
11	−1	0	0	1	50	125	110	10	2.66 ± 0.1	1.91
12	1	0	0	1	200	125	110	10	4.87 ± 0.15	7.13
13	0	−1	−1	0	125	50	20	6	0.98 ± 0.02	2.22
14	0	1	−1	0	125	200	20	6	6.51 ± 0.2	6.44
15	0	−1	1	0	125	50	200	6	1.48 ± 0.01	2.25
16	0	1	1	0	125	200	200	6	12.26 ± 0.25	11.72
17	−1	0	−1	0	50	125	20	6	2.64 ± 0.1	3.68
18	1	0	−1	0	200	125	20	6	4.92 ± 0.1	3.96
19	−1	0	1	0	50	125	200	6	2.19 ± 0.1	2.19
20	1	0	1	0	200	125	200	6	12.78 ± 0.2	10.77
21	0	−1	0	−1	125	50	110	1	0.72 ± 0.01	0
22	0	1	0	−1	125	200	110	1	4.25 ± 0.1	4.95
23	0	−1	0	1	125	50	110	10	2.37 ± 0.1	0.70
24	0	1	0	1	125	200	110	10	9.55 ± 0.2	9.37
25	0	0	0	0	125	125	110	6	4.86 ± 0.1	4.94
26	0	0	0	0	125	125	110	6	4.65 ± 0.1	4.94
27	0	0	0	0	125	125	110	6	5.32 ± 0.1	4.94

**Table 4:** Regression coefficients and analysis of variance results.

Source	DF	Sum of Squares	Mean Square	*F*-Value	*p*-Value
Model	14	305.43	21.82	9.94	0.0002
*x*_1_ (Microwave power)	1	58.83	58.83	26.79	0.0002
*x*_2_ (Stevoside conc.)	1	140.49	140.49	63.98	<0.0001
*x*_3_ (Curcuminoid conc.)	1	21.17	21.17	9.64	0.0091
*x*_4_ (Incubation time)	1	20.1	20.10	9.15	0.0106
*x*_1_ *x*_2_	1	14.21	14.21	6.47	0.0257
*x*_1_ *x*_3_	1	17.26	17.26	7.86	0.0159
*x*_1_ *x*_4_	1	0.62	0.62	0.28	0.6037
*x*_2_ *x*_3_	1	6.89	6.89	3.14	0.1019
*x*_2_ *x*_4_	1	3.33	3.33	1.52	0.2417
*x*_3_ *x*_4_	1	2.25	2.25	1.02	0.3314
*x*_1_^2^	1	1	0.06	0.8053	0.0214
*x*_2_^2^	1	1	0.30	0.5959	0.3654
*x*_3_^2^	1	1	0.33	0.5778	0.3711
*x*_4_^2^	1	1	5.87	0.0322	0.2707
Regression	14	305.83	21.82	9.94	0.0002
Residual	12	26.35	2.20		
Lack of fit	10	26.12	2.61	22.24	0.4183
Pure error	2	0.23	0.12		
Corrected total	26	331.78			

*R*^2^ = 0.9206; adjusted-R^2^ = 0.8279; adequate precision = 11.5.

## Data Availability

Data are contained within the article.
